# Supplementary material for: Comprehensive characterization of single-cell full-length isoforms in human and mouse with long-read sequencing
Source: Genome Biol. 2021 Nov 11;22:310. doi: 10.1186/s13059-021-02525-6 (PMC8582192; doi:10.1186/s13059-021-02525-6)
Supplement: Supplementary file 2 — Additional file 2. Supplementary Figures. [file 13059_2021_2525_MOESM2_ESM.pdf]

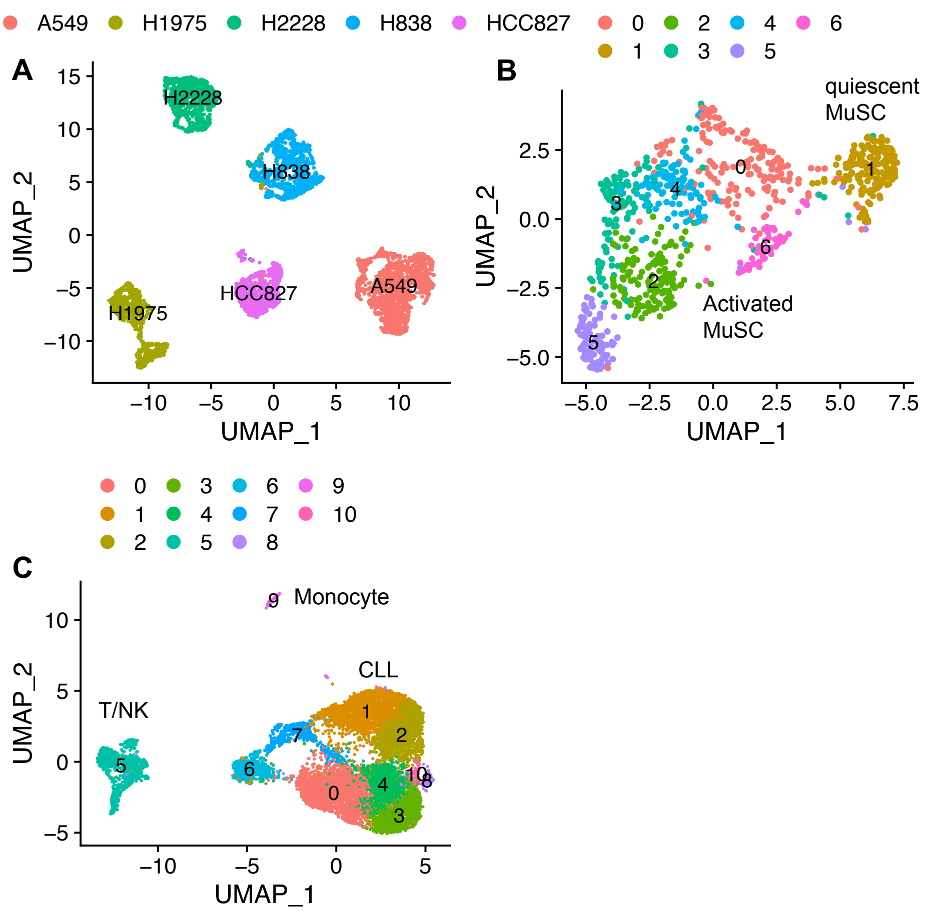

Figure S1. Dimensionality reduction (UMAP) of all samples.
UMAP visualization of cells from (A) *scmixology1* and *scmixology2* combined, (B) mouse muscle stem cells (*MuSCs*) and (C) patient *CLL2*. Cells are either colored by cell line identity (*scmixology1* and *scmixology2*) or by *Seurat* clusters and are annotated with major cell populations.

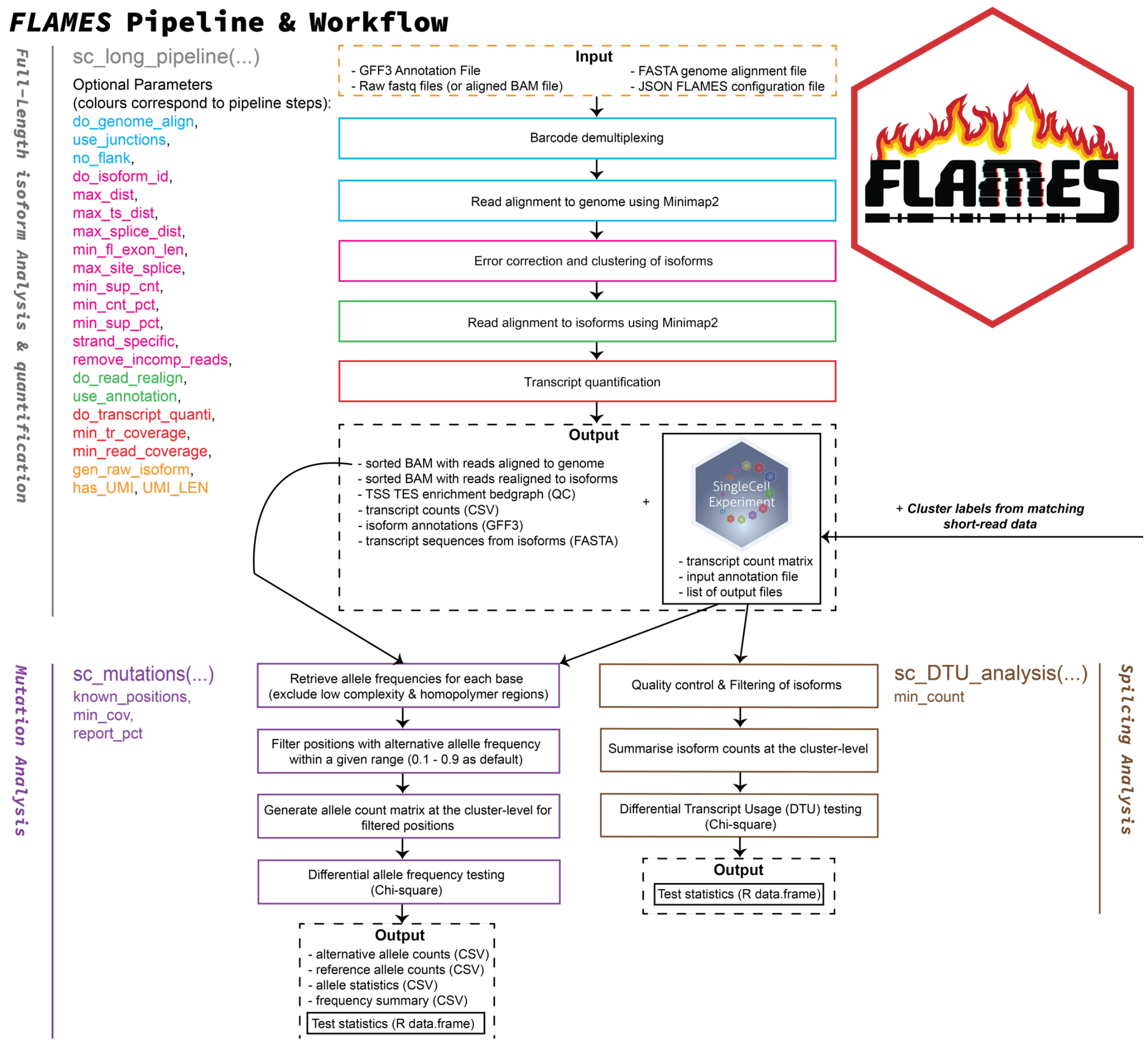

Figure S2. Overview of the major steps in the *FLAMES* analysis workflow as implemented in the R/Bioconductor package.

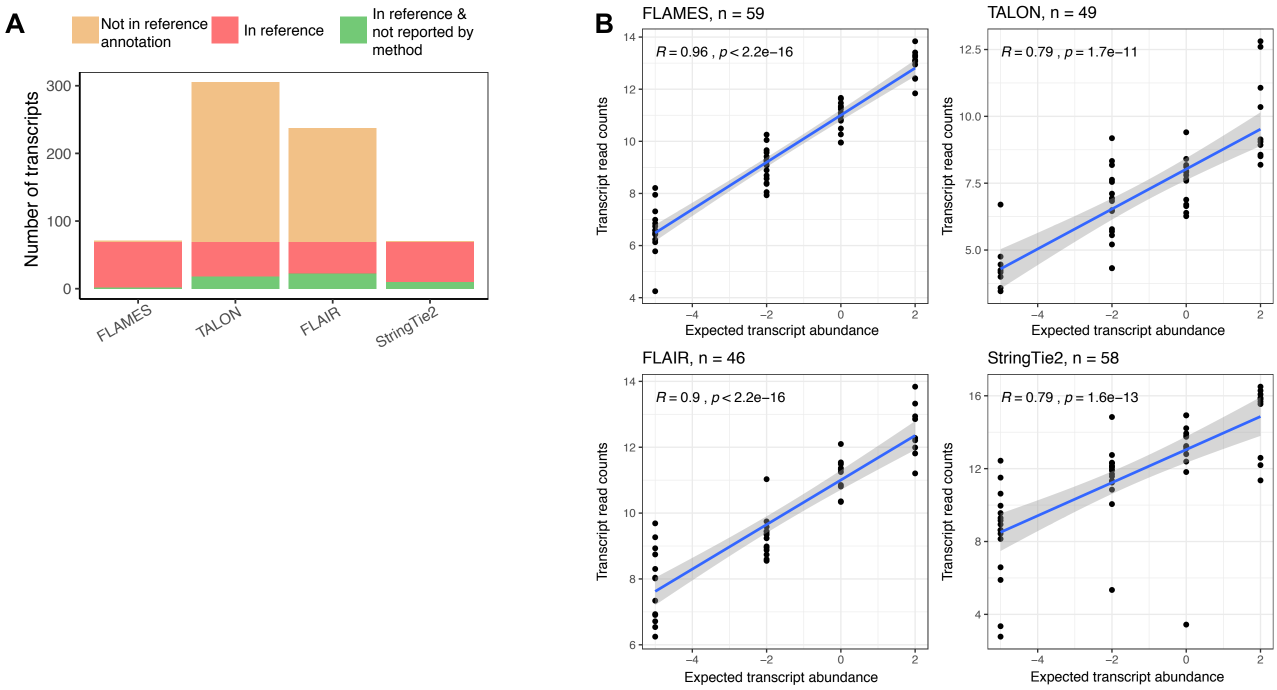

Figure S3. Comparing *FLAMES* with other methods using the SIRV dataset.
(A) Number of transcripts detected and not detected by each method, colored by whether transcript is present in the reference annotation.
(B) Scatter plot of transcript quantification where the x-axis is the expected transcript abundance based on the known concentration in the spike-in sample and the y-axis is the recovered transcript count ($\log_2$) generated by each method. The Spearman correlation coefficient (R) and *P*-value and the number of transcripts (*n*) included in the analysis are annotated in each plot. There are a total of 69 synthetic spike-in transcripts in the SIRV collection that were recovered to varying degrees by the different methods.

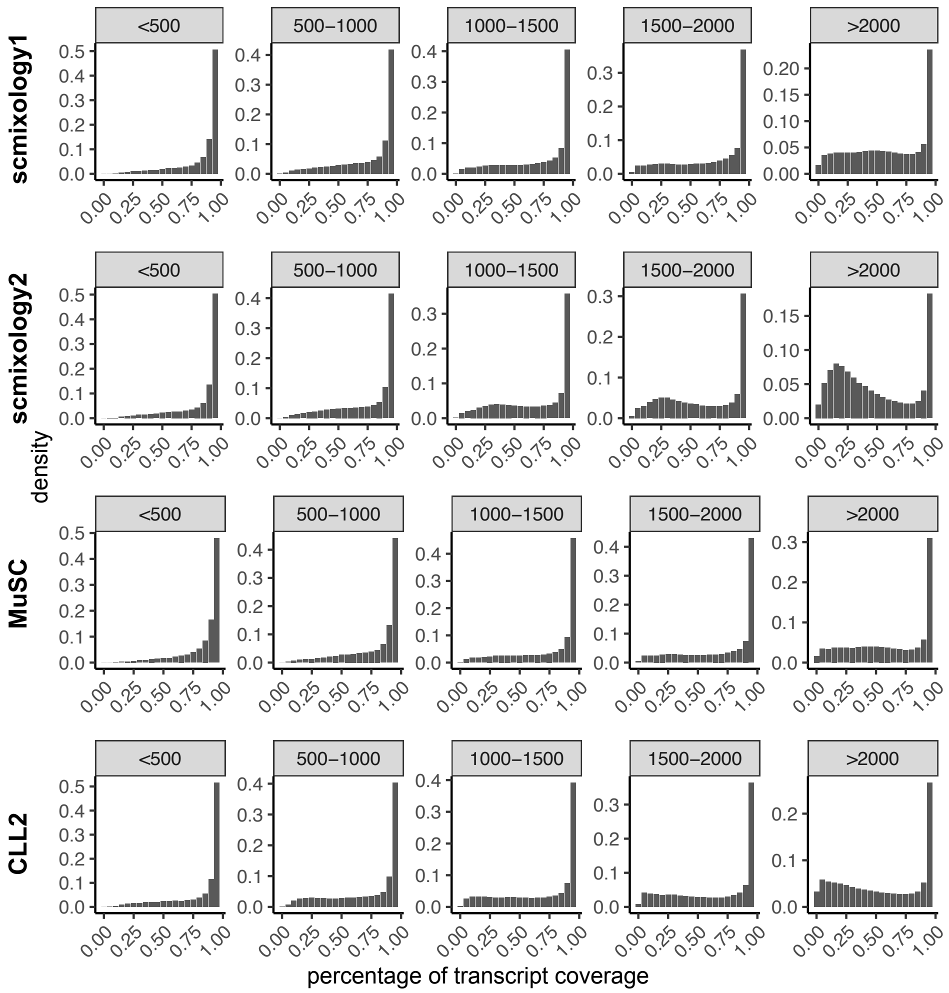

Figure S4. Percentage of transcript coverage of individual reads stratified by length.

The histogram shows the distribution of raw transcript coverage of reads aligned to the transcripts generated by *FLAMES*. Transcripts are stratified by length and divided into equal 500bp bins.

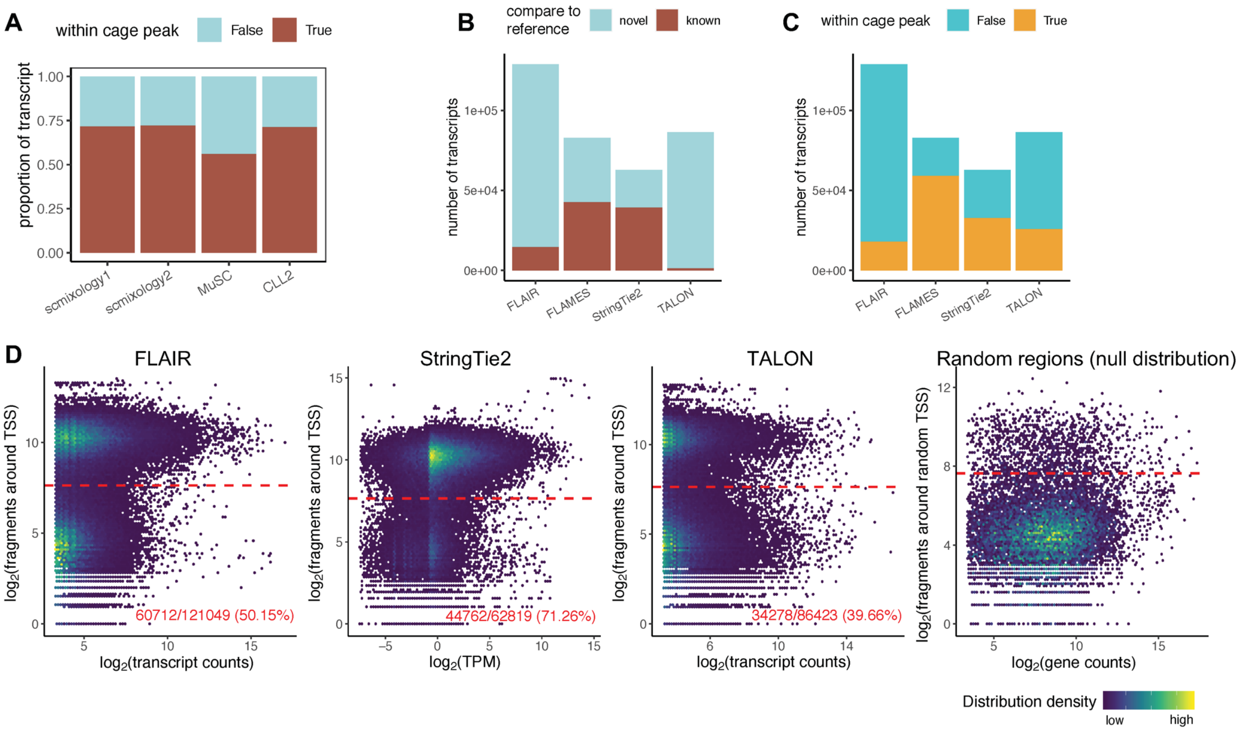

Figure S5. Comparing *FLAMES* results with database annotations and other methods.

(A) Proportion of transcript for which the TSS is within the FANTOM cage peak annotations for each sample.

(B) Number of transcripts generated by each method and the number of transcripts that match to the reference annotation.

(C) Similar to (B) and colored by the number of transcripts that fall within the FANTOM cage pack annotations.

(D) From left to right, density scatter plot showing total transcript count versus scATAC-seq read count around the TSS regions for *FLAIR*, *StringTie2* and *TALON*, together with a similar plot based on random TSS. The red line shows the threshold that separate the open chromatin and the background. The percentage shows the transcripts that have their TSSs in open chromatin regions.

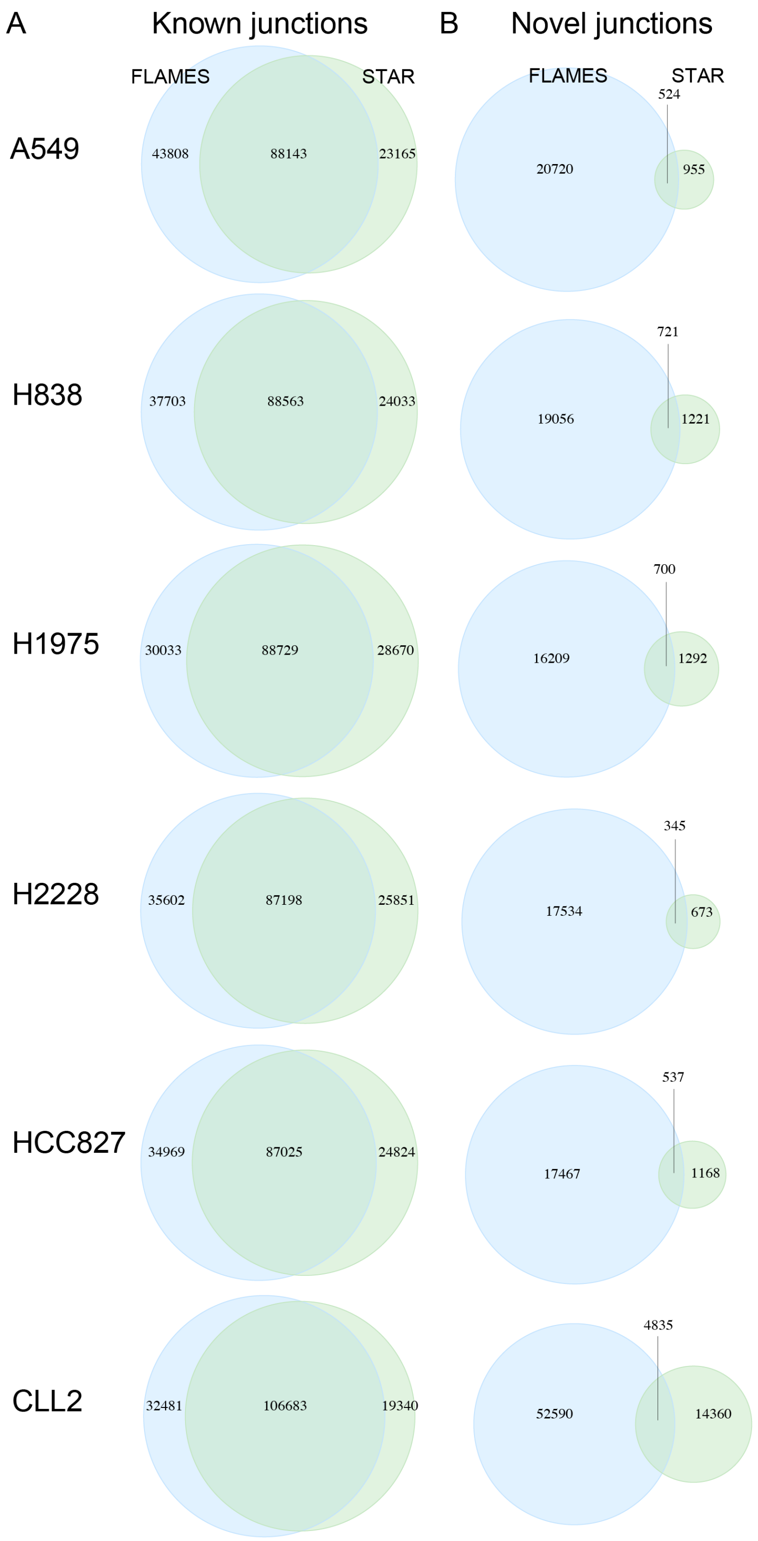

Figure S6. Venn diagrams showing the degree of junction overlap between the *FLAMES* output and bulk RNA-seq data (short-read) on the same cell lines or a matched patient sample. Results are shown per cell line for the *scmixology1* data (first 5 rows) and for *CLL2* (row 6) for known junctions (A) and novel junctions (B) for *FLAMES* in blue and *STAR* run on the relevant bulk short-read RNA-seq data in green.

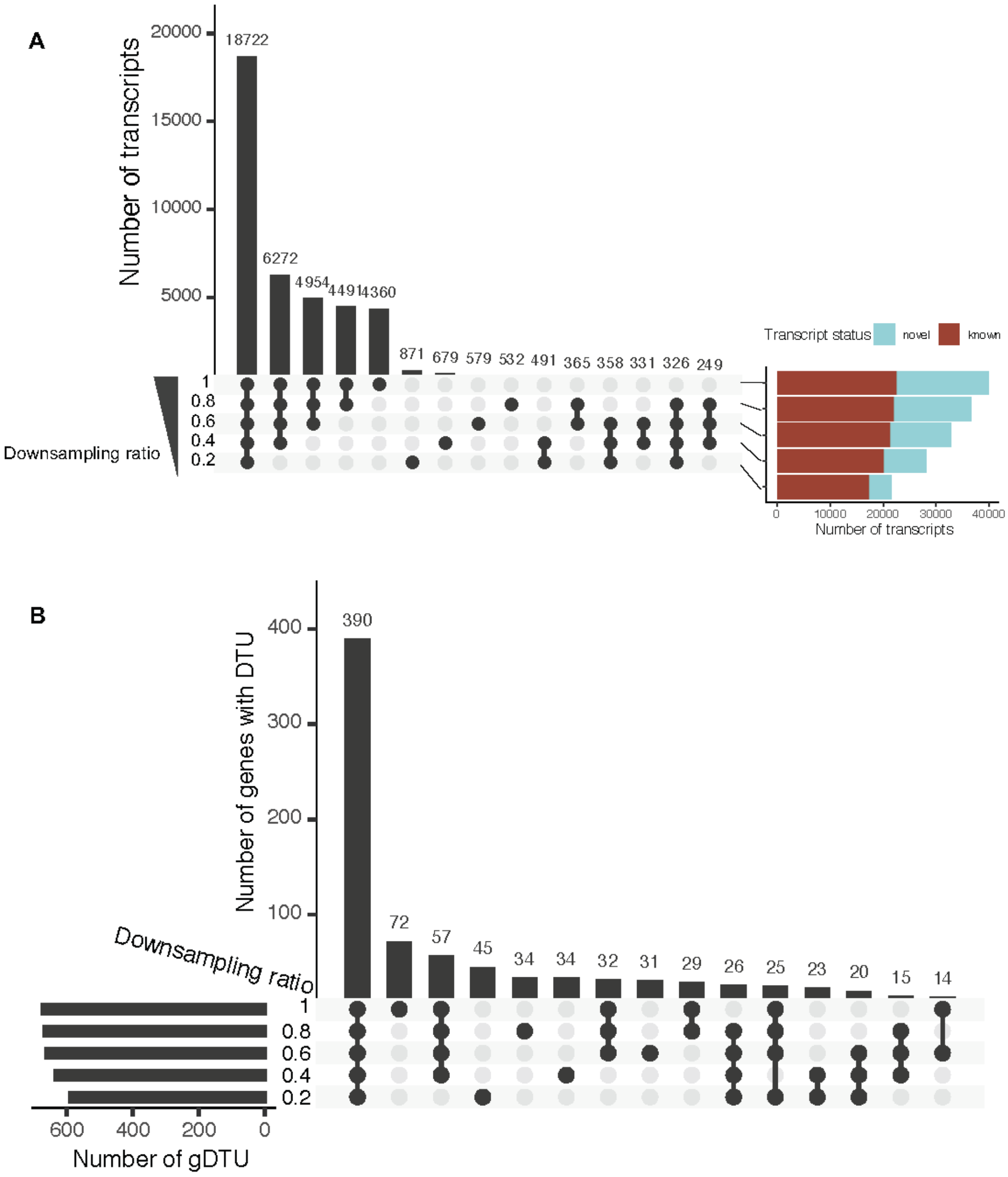

Figure S7. The effect of down-sampling on the DTU analysis.
(A) Upset plot showing the overlap of transcripts generated from down-sampling analysis of the *scmixology2* data after applying a sampling ratio from 0.2 to 0.8, annotated with the number of transcripts that match the reference annotation.
(B) Upset plot showing the overlap of genes with significant DTU on the *scmixology2* data with different down-sampling ratios (from 0.2 to 0.8).

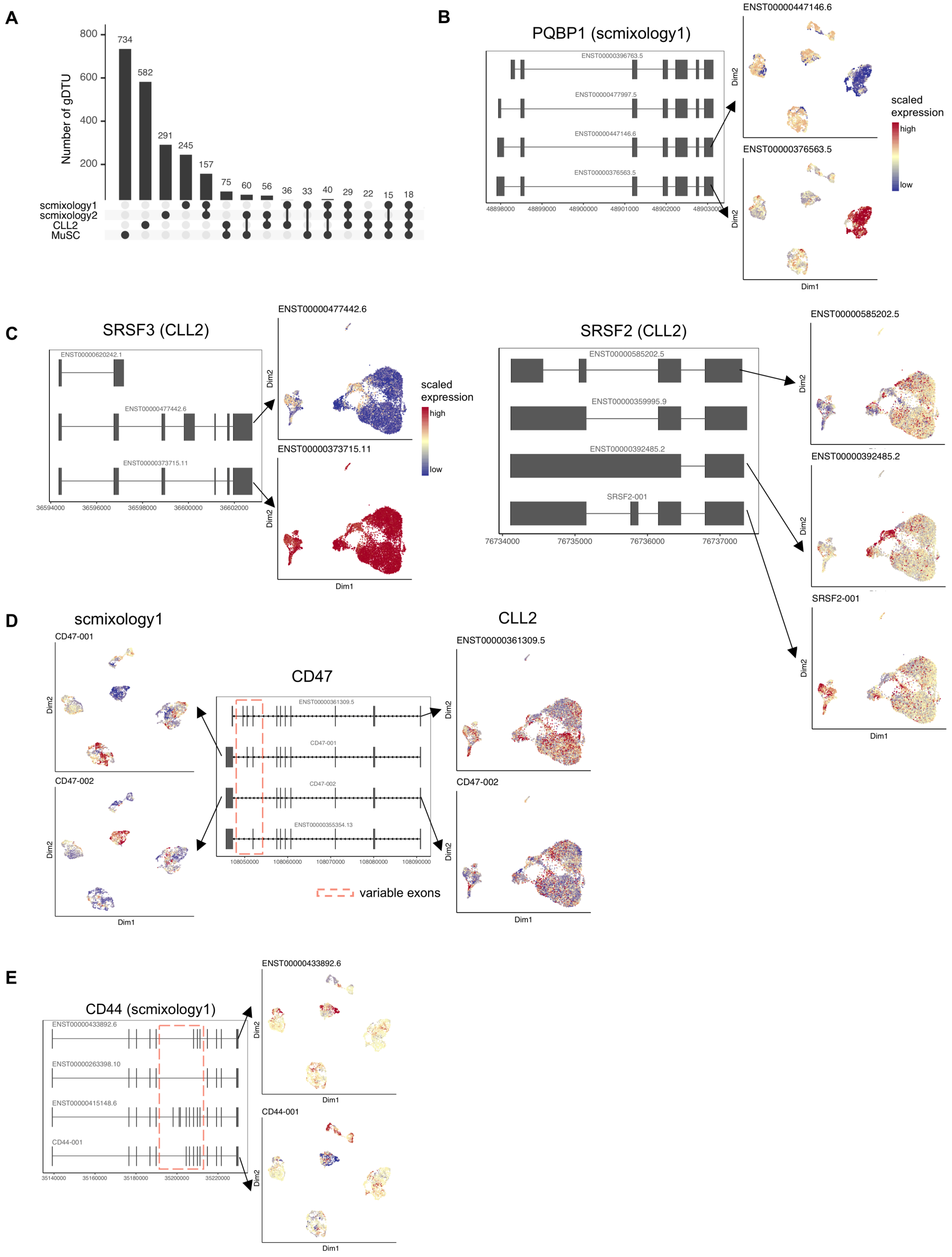

Figure S8. Genes with differential transcript usage.
(A) Upset plot shows the overlap of genes with differential transcript usage (gDTU) across different samples.

(B) Top 4 most abundant isoforms of PQBP1 in *scmixology1* and UMAP visualization colored by the expression of two isoforms with differential expression across different cell lines.
(C) Isoforms of SRSF3 in *CLL2*, with UMAP visualization colored by two isoforms with differential expression across different clusters
(D) Top 4 most abundant isoforms of CD47 in *scmixology1* and *CLL2*, together with UMAP visualization of expression of selected transcripts. Exons with alternative splicing was highlighted.
(E) Top 4 most abundant isoforms of CD44 in *scmixology1*, and UMAP visualization of transcript expression.

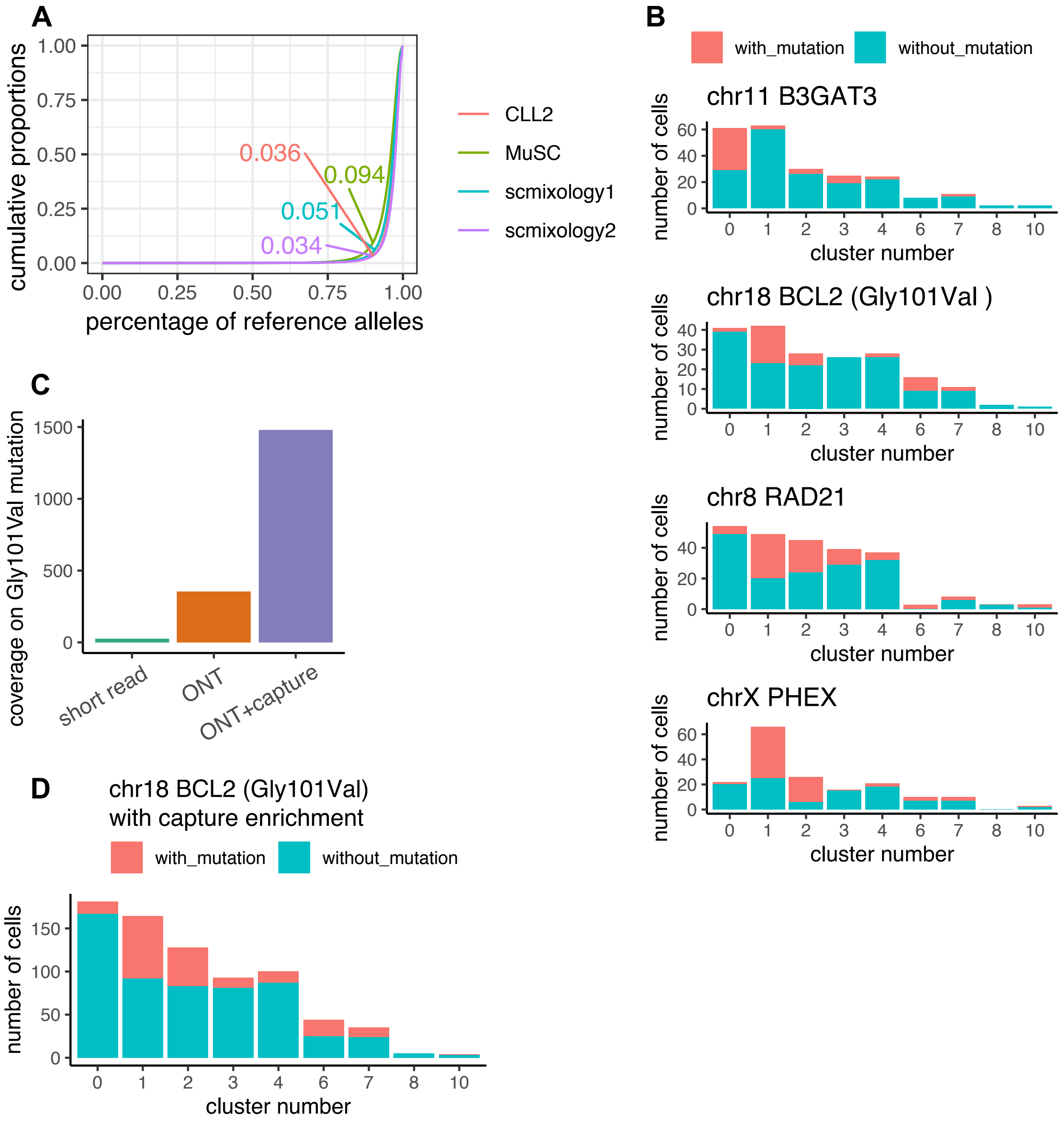

Figure S9. Identification of genomic variation from long read by *FLAMES*.
(A) Cumulative proportions of bases with certain percentage of reference alleles. The cumulative proportions of bases with 90% allele frequency were labelled on the plot, represent the percentage of reads that have lower than 90% reference allele frequency.
(B) Bar plot of four significant variants shows the distribution of cells in each cluster (x axis) that detected the mutation.
(C) The read coverage of the Gly101Val BCL2 mutation with different methods, short read stands for scRNA-seq from the same cells.
(D) Similar to (B), the bar plot of the distributions of cells with Gly101Val BCL2 mutation across clusters, with data acquired from capture enrichment (see Methods).
