# Supplementary material for: Comprehensive characterization of single-cell full-length isoforms in human and mouse with long-read sequencing
Source: Genome Biol. 2021 Nov 11;22:310. doi: 10.1186/s13059-021-02525-6 (PMC8582192; doi:10.1186/s13059-021-02525-6)
Supplement: Supplementary file 6 — Additional file 6. Review history. [file 13059_2021_2525_MOESM6_ESM.docx]

Review History

**First round of review**

**Reviewer 1**

**Were you able to assess all statistics in the manuscript, including the appropriateness of statistical tests used?**

Yes: The use of statistical tests listed in the manuscript appears to be appropriate, but I have not extensively reviewed their code and their raw data is not available for direct testing of their code, which is open-source

**Were you able to directly test the methods?**

No

**Comments to author:**

In this manuscript, the authors propose a novel bioinformatics and experimental workflows to: 1) accurately quantify both alternative splicing with mutations that arise in diseases such as cancer at a single-cell level from full-length isoform sequencing and 2) integrate/correlate disparate molecular readouts across single-cell populations. The strategy is of significant relevance to studies which need to coincidently identify: A) which cells have a mutation, 2) what genes or isoforms are associated with distinct clones versus non-malignant cells, 3) what novel associations exist between scATAC-Seq and alternative isoforms and 4) which isoforms could effectively replace doing CITE-Seq which requires extensive titration of antibodies for specific cell types.

The authors show that their software FLAMES is able to accurately detected synthetic spiked-in isoforms and cellular isoforms linked to a TSS with scATAC-Seq more accurately than other algorithms. The authors provide a few specific examples of splicing factors and cell surface proteins alternatively expressed in different CLL cell populations, including a CITE-Seq ADT with improved correlation to a specific antigen isoform or isoform switch evidenced by a distinct TSS. The authors also show evidence that mutation detection is accurate and single-cells and can distinguish clonal populations of CLL.

In the opinion of this reviewer, broad interest in this manuscript necessitates a significant analytical or methodological advance or significant novel biological insights. It may be that FLAMEs provides such a methodological advance, however, it requires a more in-depth comparison to alternative tools to highlight significant benefits over existing approaches (i.e., capabilities of the software for isoform and mutation detection, novel insights from multiple molecular modalities [including improved isoform quantification with the aid of matching standard 10x transcript UMI data], ease-of-use, sensitivity and specificity for both new and existing datasets using diverse reasonable benchmarks vs. other algorithms). In terms of biological insights, most biological observations are global (novel vs. novel events, splice-event types detected from Nanopore), but new insights into cancer associated splicing events/isoforms are not shown (i.e., impact of intron retention within distinct genes or pathways with clear relationships to patient outcome/therapy).

Primary Concerns

1. The authors show that FLAMES has improved accuracy to detected spiked in synthetic transcripts. These results appear to be due to FLAMES's conservative alignment to known transcripts. It is not clear if these synthetic transcript references exist in the FLAMES database, lending towards biased detection. While the use of synthetic isoforms for validation is useful, it does not negate the possibility that other algorithms are more sensitive to identify novel or known disease isoforms in different contexts.

2. Along this line, if FLAMES was applied to a malignant bulk full-length isoform sample, with known splicing factor mutations, such as SF3B1 (PMCID: PMC7080807), what is its sensitivity to detect prior validated cancer-specific splice events/isoforms of which dozens of splicing-events are well-described in the literature?

3. The improved correlation of isoforms with CITE-Seq ADTs would be very exciting and an important observation for the field, to highlight the frequent discordance of ADT and gene expression from CITE-Seq. However, as only one example is given, it is unclear whether this is a broad phenomena. It is worth noting that for the example shown, this 1306aa transcript is actually CD45RABC, which does not correspond to the CD45RA-ADT (1211aa long). This factor negates the claims made on page 12 line 13-27, but explains the inconsistency. The authors used a 17 ADT Total-Seq C panel from Biolegend, however little data is shown. It is concerning as the authors used frozen CLL peripheral blood samples and rested those cells in 37 degrees for just 2 hours before capture. It is not clear whether surface markers are present on cell surface with this protocol (not internalized). Please show the expression distribution of all ADTs.

4. For mutation detection, it is unclear whether FLAMES performs all of the major analytical steps, including exclusion of homopolymer regions. As other analysis workflows exist for variant detection from Nanopore data, how does FLAMES workflow compare to these (speed, accuracy, integration with isoform detection)?

Minor Concerns

5. The primary experimental innovation is subsampling of GEMs to get deeper Nanopore sequencing per cell. The raw reads (sequence) from the Nanopore do not appear to be deposited to enable re-analysis from the raw data. Defined cell clusters do not appear to be provided in GEO or as a supplemental table. Further, it is not clear that the CITE-Seq ADTs have been deposited or counts data made available. Accession for EGA should be provided.

6. Additional detail is required in Fig. S2's legend to indicate that these are 69 synthetic spike-in isoforms.

7. The authors note that: "We frequently found complex splicing changes between the two most abundant isoforms of a gene, suggesting the inadequacy of studying individual splicing junctions via short reads." While this is an agreed benefit of long-read sequencing, it is not clear this is clearly reflected in any figures or analyses, on a global basis.

8. Can the differential isoform analysis in FLAMES be validated using PSI-based splicing approaches on the same data or similar data?

9. How does the detection of genomic variants (point mutations, structural variation) with FLAMES and the experimental protocol used compare to recent reported methods such as GOT (e.g., Nam et al. 2019 Nature v.571, pg.355-360)?

10. In the scRNA-seq cell isolation and library preparation Methods, the authors note that they increase post-GEM RT incubation for two hours to "increase reverse transcription of longer transcripts". It is hard to believe that 1-hour is enough for any commercial reverse transcriptase to produce longer templates using bulk RNA. The authors did not mention the extension time for cDNA amplification which could be a critical determinant of cDNA transcript size. To support the claims made, please show the fragment distribution graph of amplified cDNA (e.g., BioAnalyzer).

11. The authors claim this modified protocol could provide a "cost-effective" approach compared to previous methods. As shown in Figure 1C, half of the Nanopore reads are wasted on TSO artifacts that do not contain cell barcode, reviewer acknowledge this is a known pitfall however several approaches have been developed to remove TSO, which needs to be mentioned.

12. The authors appear to mean reads rather than median UMI when referring to 30k UMI per cell in Fig. 1D (30k UMI per cell barcode for 10x Chromium is unheard of).

13. How did the authors deal with technology associated batch effects in the scmixology dataset (version 2 and version 3 chemistries).

14. The rationale for using scPipe for the MuSC and Scmixology datasets and CellRanger for the CLL dataset for estimating gene counts is not clear. This may result in distinct UMI estimates for Fig. 2C.

**Reviewer 2**

**Were you able to assess all statistics in the manuscript, including the appropriateness of statistical tests used?**

Yes: The statistics in the manuscript are not too complicated

**Were you able to directly test the methods?**

No

**Comments to author:**

The paper by Tian et al. constitutes a contribution to the emerging field of single cell long - read sequencing. Single cell long read sequencing will crucially contribute to the completion of the annotation of the set of genes and transcripts in the human genome, since it will eventually provide full length transcript sequences monitored in the diversity of the cell types in the human body. Although we are still far from this goal, the paper of Tian et al., together with a number of recent papers, are helping to set the path.

Maybe the main contribution of Tian et al. is that by subsampling a population of single cells, they achieve sequencing depth which his comparable to short read sequencing, and therefore they show that long read (single-cell) sequencing can be used not only for transcript discovery, but also for transcript quantification.

The approach developed by the authors is a combination of existing methods: 10x chromium single cell kit + Smart-seq3 protocol to prepare full-length cDNA (as already published) and ONT for long read sequencing, rather than a development of a new method, but they show that their approach can be successfully compared to other existing experimental and computational approaches.

Overall the paper is clearly written and well organized, and the data allows for some interesting findings regarding biology, specially regarding alternative splicing AS (i.e. the most abundant AS events are complex splicing changes, which are difficult to capture with short read sequencing. I do not have serious technical issues with the manuscript, but I have the following issues that the authors may want to take into account:

1. The authors write "the throughput of current long-read sequencing platforms is still not comparable to short-read platforms and the per-base accuracy is also lower". PacBio Sequel II HiFI reads now match HiSeq in base accuracy

2. The authors should comment on what gives FLAMES such a striking advantage over well-established tools. According to Fig S2 it reaches ~100% accuracy on SIRVs using ONT direct RNA data. This is really striking, but sensitivity and precision values should be provided as a supplementary table or the like.

3. Genome-mapped and SIRVome-mapped reads should undergo the same processing steps (as explained page 19, lines 58 onward for the former). More specifically, do the SIRV reads also undergo the "two-pass" polishing steps mentioned on page 20?

4. Figure S3. If I understand the figure, there is a strong 3'bias, which suggests that the transcripts may be rather short. Also, the bias is quite different for scimixology1 and scimixology2. Any hypothesis to explain this?

5. Other than the comparison with the SIRVs there is little characterization of the transcripts sequenced: the distribution of transcript lengths, the proportion of full lengh transcripts, the proportion of known and novel transcripts—although some numbers are given in the text, we do not know how many novel transcripts have been discovered through this work--, what fraction novel transcripts map to intergenic regions, and of those whether there is any indication of coding potential (for instance, using PhyloSCF). For annotated transcripts do we get novel exons (any potentially novel protein coding exon?) Also, it is not clear what transcript annotation was employed.

6. I also think that it would be interesting to see how many novel transcripts do we get per cell, and whether there is some saturation as we add novel cells.

7. More details on the FLAIR runs are needed. For example, what module was used, "align", "correct", "collapse", etc., or all of them? I am not 100% familiar with FLARI or TALON, but it seems to me that skipping the collapse step would give them an unfair disadvantage when compared to other tools that collapse by default (such as StringTie2 and, I guess, FLAMES), both in terms of transcript model and TSS accuracy (truncated reads have a much higher probability of being collapsed into a full-length one).

8. The "two-pass" polishing step looks very convoluted to me (to be honest I'm not sure I fully understand it), and it's unclear what advantage it brings. The authors should provide a comparison of accuracy of transcript models with and without it, for example on SIRVs

9. page 9, line 44: "Around 80% of genes can be expressed as multiple isoforms. " Please provide a reference for this statement

10. Figure 2F. "The distribution of isoforms expression is skewed with only a few abundant transcripts dominantly expressed for most genes." The authors should cite https://pubmed.ncbi.nlm.nih.gov/23815980/

11. The data on the different allele frequency of a few genes correlating with CLL expression clusters is quite interesting, but it is sort of frustrating that it is not bringing new biological insights. Maybe the authors could compare with the available DNA and matched bulk RNAseq data to see whether they could validate the sc results.

**Authors Response**

**Point-by-point responses to the reviewers’ comments:**

In this manuscript, the authors propose a novel bioinformatics and experimental workflows to: 1) accurately quantify both alternative splicing with mutations that arise in diseases such as cancer at a single-cell level from full-length isoform sequencing and 2) integrate/correlate disparate molecular readouts across single-cell populations. The strategy is of significant relevance to studies which need to coincidently identify: A) which cells have a mutation, 2) what genes or isoforms are associated with distinct clones versus non-malignant cells, 3) what novel associations exist between scATAC-Seq and alternative isoforms and 4) which isoforms could effectively replace doing CITE-Seq which requires extensive titration of antibodies for specific cell types.

The authors show that their software FLAMES is able to accurately detected synthetic spiked-in isoforms and cellular isoforms linked to a TSS with scATAC-Seq more accurately than other algorithms. The authors provide a few specific examples of splicing factors and cell surface proteins alternatively expressed in different CLL cell populations, including a CITE-Seq ADT with improved correlation to a specific antigen isoform or isoform switch evidenced by a distinct TSS. The authors also show evidence that mutation detection is accurate and single-cells and can distinguish clonal populations of CLL.

In the opinion of this reviewer, broad interest in this manuscript necessitates a significant analytical or methodological advance or significant novel biological insights. It may be that FLAMEs provides such a methodological advance, however, it requires a more in-depth comparison to alternative tools to highlight significant benefits over existing approaches (i.e., capabilities of the software for isoform and mutation detection, novel insights from multiple molecular modalities [including improved isoform quantification with the aid of matching standard 10x transcript UMI data], ease-of-use, sensitivity and specificity for both new and existing datasets using diverse reasonable benchmarks vs. other algorithms). In terms of biological insights, most biological observations are global (novel vs. novel events, splice-event types detected from Nanopore), but new insights into cancer associated splicing events/isoforms are not shown (i.e., impact of intron retention within distinct genes or pathways with clear relationships to patient outcome/therapy).

Primary Concerns

1. The authors show that FLAMES has improved accuracy to detected spiked in synthetic transcripts. These results appear to be due to FLAMES's conservative alignment to known transcripts. It is not clear if these synthetic transcript references exist in the FLAMES database, lending towards biased detection. While the use of synthetic isoforms for validation is useful, it does not negate the possibility that other algorithms are more sensitive to identify novel or known disease isoforms in different contexts.

*Response: We thank the reviewer for their interest in our work and constructive feedback. The strategy FLAMES uses for its transcript-level analysis of the sequins is to first align all reads to the sequin reference chromosomes, within which the artificial transcripts are contained. This part of the analysis doesn’t require any specific knowledge of the transcripts and is analogous to what would happen when aligning reads to a reference genome from any species (such as human and mouse genomes which were used for the other datasets in our manuscript). Next, we assemble the isoforms from the alignment results to obtain draft isoforms. The draft isoforms are compared against the reference annotation to annotate them and correct possible errors. The final step is to realign the reads to the transcriptome defined in the previous step to allow an accurate quantification of the different isoforms present in the sample. This approach is semi-supervised, as the reference annotation is used and extended upon depending on the isoforms present. It is worth noting that all methods compared make use of the reference annotation to help with isoform detection, and were supplied with the sequin annotation, so the comparison is fair in this regard. We agree that the results obtained using these artificial isoforms doesn’t guarantee better performance on data obtained from real experimental samples, but the ground-truth available does provide an independent means of comparing the results from the different methods, which is a reasonable starting point.*

*To assess the results further, we examined the junction reads obtained in bulk short-read RNA-seq from the same 5 cell lines used in the scmixology and the patient sample (CLL2) using STAR and compared them to those obtained using FLAMES on the single-cell data. These show relatively high agreement for annotated junctions (new Figure S6A) and less overlap for novel ones (new Figure S6B). The detection of novel junctions is where we would expect the long-read data to outperform the short-read results, as most long-reads have the potential to contribute information on junctions, while in the short-read data only a smaller subset of reads map to junctions.*

*For the cell line mixture dataset we also compared the transcription start site (TSS) assignment, which can be roughly inferred from the scATAC-seq data generated from the same population. This analysis shows that FLAMES (Figure 1F) has a higher level of greater agreement compared with other tools (Figure S5D).*

*Taken together, these results give us confidence that our method is performing well. Nonetheless, we are committed to further benchmarking of FLAMES, and recently provided its output on the reference samples from the Long-read RNA-seq Genome Annotation Assessment Project (LR-GASP, https://www.gencodegenes.org/pages/LRGASP/) to support the systematic method comparisons planned by this consortia and further advance this field. We have added a sentence on the importance of further benchmarking to the Discussion:*

*‘There is also a pressing need for further systematic benchmarking of long-read isoform analysis methods, which is currently being pursued by the Long-read RNA-seq Genome Annotation Assessment Project (LR-GASP, https://www.gencodegenes.org/pages/LRGASP/).’*

2. Along this line, if FLAMES was applied to a malignant bulk full-length isoform sample, with known splicing factor mutations, such as SF3B1 (PMCID: PMC7080807), what is its sensitivity to detect prior validated cancer-specific splice events/isoforms of which dozens of splicing-events are well-described in the literature?

*Response: We thank the reviewer for this suggestion. As the manuscript is principally concerned with applying long-read sequencing to single-cell data, we applied the protocols described in this paper on samples from CLL patients with and without SF3B1 mutations and a healthy donor sample and examined the splicing changes. Our analysis using FLAMES reveals enhanced novel isoform detection in SF3B1 mutated samples. The heatmap below shows novel isoform expression of different genes in the rows across different samples in the columns. Samples from different conditions with wild-type SF3B1 (including the healthy donor sample) were pooled together. We found genes that appear in previous studies such as ZNF548, ZNF91 and ERGIC3, and new genes such as the ribosomal genes (RPL24, RPL27A, RPS10, RPS12) and SF3A2 which is also a splicing factor. TPT1 has a novel isoform which is only expressed in samples with an SF3B1 mutation (shown in the bar plot where blue represents the major isoform and red represent the novel isoform). This novel isoform has two splice site differences in the first and last exon (highlighted in the isoform structure diagram beside the bar plot). This result highlights the benefit of using long-read sequencing for isoform discovery since it is difficult for short-read sequencing to link such distal splice site alterations.*

*The data from these patients and their analysis is destined for a follow up study led by our collaborators, and will be made available from EGA in due course. Nonetheless, they demonstrate the power of this approach to explore splicing changes in scRNA-seq data.*

3. The improved correlation of isoforms with CITE-Seq ADTs would be very exciting and an important observation for the field, to highlight the frequent discordance of ADT and gene expression from CITE-Seq. However, as only one example is given, it is unclear whether this is a broad phenomena. It is worth noting that for the example shown, this 1306aa transcript is actually CD45RABC, which does not correspond to the CD45RA-ADT (1211aa long). This factor negates the claims made on page 12 line 13-27, but explains the inconsistency. The authors used a 17 ADT Total-Seq C panel from Biolegend, however little data is shown. It is concerning as the authors used frozen CLL peripheral blood samples and rested those cells in 37 degrees for just 2 hours before capture. It is not clear whether surface markers are present on cell surface with this protocol (not internalized). Please show the expression distribution of all ADTs.

*Response: The CD45RA antibody detects RABC, RAB and RA isoforms (see table below). Following stimulation, naive cells lose CD45RA and gain CD45RO expression to become memory cells. Most T cells in the CLL sample are memory T cells and express CD45RO, which is not detected by the CD45RA antibody.*

*Antibody	Isoforms Detected*

*CD45	ABC, AB, BC, B, O*

*CD45RA	ABC, AB, A*

*CD45RB	ABC, AB, BC, B*

*CD45RO	O*

*In light of this, we believe that the claim we have made that the reviewer highlights above is still valid and have left it as is. CD45 is the only protein in our data that has multiple antibodies targeting different isoforms, and its isoforms and functions have been well studied. So We only correlated isoform expression and protein level on CD45. We hope to have more such examples in the future experiments where I would have a larger antibody panel.*

*The primary CLL cell protocol is well established in the lab of our collaborators (Thijssen et al. Blood 2016; Blombery et al., Cancer Discovery 2019). The viability of CLL cells was 83% (PI negative) after 2 hours of culture and staining. The 17 Total-Seq C antibodies were validated by secondary staining with an oligo-conjugated with APC and measured by flow cytometry.*

*A violin plot of the log-transformed expression of the ADTs, which shows a good range of expression values for each antibody, is presented below.*

4.	For mutation detection, it is unclear whether FLAMES performs all of the major analytical steps, including exclusion of homopolymer regions. As other analysis workflows exist for variant detection from Nanopore data, how does FLAMES workflow compare to these (speed, accuracy, integration with isoform detection)?

*Response: We apologize that the approach taken by FLAMES for mutation analysis wasn’t sufficiently clear in the Methods. We have added a new supplementary figure (Figure S2) to clarify the various steps that FLAMES manages. In summary, after excluding homopolymer and low complexity regions, allele frequencies for each base are retrieved and positions with an alternative allele frequency within a given range (0.1-0.9 by default) are retained. An allele count matrix at the cluster-level is then generated for the filtered positions, and a test for differential allele frequency is carried out using a chi-square test.*

*There is definitely scope to modularize FLAMES to use nanopore-specific variant callers which we will consider as future work. This is not the main purpose of our pipeline however, rather it is to exploit the single-cell nature of the data and stratify allele counts by cluster to look for mutation patterns that vary between them. It also aims to screen out variants that are either germline or noise that have consistent allelic imbalances across clusters. To the best of our knowledge this extra component is not available in current tools, as mutation analysis is not yet a routine part of a scRNA-seq analysis.*

Minor Concerns

5.	The primary experimental innovation is subsampling of GEMs to get deeper Nanopore sequencing per cell. The raw reads (sequence) from the Nanopore do not appear to be deposited to enable re-analysis from the raw data. Defined cell clusters do not appear to be provided in GEO or as a supplemental table. Further, it is not clear that the CITE-Seq ADTs have been deposited or counts data made available. Accession for EGA should be provided.

*Response: Raw data are available from GEO for the cell line and mouse data sets, which will allow others to analyse these data (a recent example of reuse of the cell line data can be found in Davidson et al. https://www.biorxiv.org/content/10.1101/2021.04.26.441398v2 who identify fusion transcripts (including novel 3 gene fusions) in the single-cell long-read data). The counts are available for these data sets from GEO and GitHub. The cluster information is available from our GitHub repository that supports this analysis (https://github.com/LuyiTian/FLTseq_data/tree/master/data). The data from CLL2 (counts for transcripts / genes / ADT) are available in EGA under accession number EGAS00001005597. We have added extra information to the ‘Availability of data and materials’ section to clarify what data is available and where to access it.*

6.	Additional detail is required in Fig. S2's legend to indicate that these are 69 synthetic spike-in isoforms.

*Response: We have added this extra information to the legend for this Figure (now S3, previously S2).*

7.	The authors note that: "We frequently found complex splicing changes between the two most abundant isoforms of a gene, suggesting the inadequacy of studying individual splicing junctions via short reads." While this is an agreed benefit of long-read sequencing, it is not clear this is clearly reflected in any figures or analyses, on a global basis.

*Response: This statement refers to the data presented in Figure 2G which is discussed in the Results.*

8.	Can the differential isoform analysis in FLAMES be validated using PSI-based splicing approaches on the same data or similar data?

*Response: We agree that this would be a good basis for the comparison of differential isoform results. To do a thorough job of this, we are planning a bulk RNA-seq benchmarking data set that has matched samples run on both Nanopore and Illumina sequencing platforms with 2 experimental groups. Data generation has begun for this project and the subsequent analysis to compare the differential isoform results in this manner is left as future work. We envisage that more sophisticated methods for testing may perform better than the simple chi-square test we currently have in FLAMES, and will add additional methods as options based on the results of this benchmark analysis in the future. We have also added a down-sampling analysis (Figure S7) to assess how reliable the differential transcript usage (DTU) results are when the number of reads used as input to FLAMES varies from 20% up to 80% of the original number of long reads from the scmixology2 data. As one would expect, we do find fewer isoforms when we start with fewer reads, but nonetheless, the majority of genes detected to have DTU (58%) are found in common between the various subsampling analyses, which provides some extra evidence that the simple approach we have taken gives reasonable results.*

9.	How does the detection of genomic variants (point mutations, structural variation) with FLAMES and the experimental protocol used compare to recent reported methods such as GOT (e.g., Nam et al. 2019 Nature v.571, pg.355-360)?

*Response: Thank you for pointing out this reference, which we have added mention of in the Discussion. Compared to our approach, GoT uses primers and is not coupled with long-read sequencing. GoT can only detect variants that primers are designed for, while our approach is unbiased, and transcripts of interest can be enriched from full-length cDNA libraries afterwards. Although we note that GoT has recently been adapted for long-read sequencing according to a presentation by Dr Dan Landau at London Calling 2021, no primary reference for this work is available as yet as far as we know.*

10.	In the scRNA-seq cell isolation and library preparation Methods, the authors note that they increase post-GEM RT incubation for two hours to "increase reverse transcription of longer transcripts". It is hard to believe that 1-hour is enough for any commercial reverse transcriptase to produce longer templates using bulk RNA. The authors did not mention the extension time for cDNA amplification which could be a critical determinant of cDNA transcript size. To support the claims made, please show the fragment distribution graph of amplified cDNA (e.g. BioAnalyzer).

*Response: Below are the TapeStation profiles for scmixology2 cDNA (45 min RT and 1 min extension for cDNA amplification, panel A) and CLL2 (2 hour RT and 3 min extension for cDNA amplification, panel B) which indicate a slight increase in fragment length was obtained from lengthening RT time in the CLL2 sample. This is not an ideal comparison though, as the CLL2 cDNA could well be longer due to the presence of different cell types in this sample compared to the scmixology sample. In light of this, we have modified the methods as follows:*

*‘Briefly we followed the standard 10x Genomics user guide, with RT time increased to 2 hours in the CLL sample only to potentially increase the reverse transcription of longer transcripts.’*

11.	The authors claim this modified protocol could provide a "cost-effective" approach compared to previous methods. As shown in Figure 1C, half of the Nanopore reads are wasted on TSO artifacts that do not contain cell barcode, reviewer acknowledge this is a known pitfall however several approaches have been developed to remove TSO, which needs to be mentioned.

*Response: We agree, that reducing TSO artifacts may increase the fraction of usable reads and have added mention of Lebrigand et al. (2020) who propose a solution to this to the Discussion:*

*‘... we demonstrate that a similar per-cell sequencing level can be achieved to short read technologies at a much lower cost than other methods because only a subset of the cell population undergoes nanopore sequencing. As library preparation artefacts can cause a substantial fraction of reads to lack barcodes (40-60% in this study), enriching for full-length cDNAs that contain barcodes as proposed in Lebrigand et al. has the potential to further enhance the efficiency of sequencing.’*

*Future improvements to FLAMES will include reporting of the proportion of reads that are likely TSO errors as a QC metric, so that the experimenter is aware of the extent of this issue.*

12.	The authors appear to mean reads rather than median UMI when referring to 30k UMI per cell in Fig. 1D (30k UMI per cell barcode for 10x Chromium is unheard of).

*Response: We do in fact mean UMIs - the high number reflects the fact that these cells are transcriptionally very active. This is consistent with earlier runs on the same cell lines across multiple scRNA-seq protocols, including 10x (refer to Supp Figure 2B from Tian et al. (2018) bioRxiv, https://www.biorxiv.org/content/10.1101/433102v1.full.pdf which is shown below). In this benchmarking experiment, all datasets involved different cell mixing strategies using cells from the same scmixology lines - sc_10X is an earlier 10X run which has a median UMI count per barcode of ~ 100k.*

13.	How did the authors deal with technology associated batch effects in the scmixology dataset (version 2 and version 3 chemistries).

*Response: Batch effects were not an issue for our analysis, as we did not merge the scmixology data from different chemistries. It certainly could be a problem though, especially when the cell processing and 10x runs for different samples are performed at on separate occasions and the goal is to analyse between samples. If we did need to combine data from different batches, we would consider using methods like MNN (Mutual Nearest Neighbours), which have been found to perform well in previous benchmarking studies (e.g. Tian et al. (2019) Nat Methods) and are applicable assuming some common cell types are present between datasets. Processing the data together in the isoform analysis step of FLAMES is another possibility that we will explore further as we get access to more multi-sample datasets.*

14. The rationale for using scPipe for the MuSC and Scmixology datasets and CellRanger for the CLL dataset for estimating gene counts is not clear. This may result in distinct UMI estimates for Fig. 2C.

*Response: This choice was due to scPipe’s current limitation of not handling the ADT sequences and therefore not being able to create an ADT matrix. We aim to add this capability to future versions of scPipe along with support for scATAC-seq and sc-multiome data. This extension is beyond the scope of the current paper though.*

Point-by-point responses to Reviewer #2:

The paper by Tian et al. constitutes a contribution to the emerging field of single cell long - read sequencing. Single cell long read sequencing will crucially contribute to the completion of the annotation of the set of genes and transcripts in the human genome, since it will eventually provide full length transcript sequences monitored in the diversity of the cell types in the human body. Although we are still far from this goal, the paper of Tian et al., together with a number of recent papers, are helping to set the path.

Maybe the main contribution of Tian et al. is that by subsampling a population of single cells, they achieve sequencing depth which his comparable to short read sequencing, and therefore they show that long read (single-cell) sequencing can be used not only for transcript discovery, but also for transcript quantification.

The approach developed by the authors is a combination of existing methods: 10x chromium single cell kit + Smart-seq3 protocol to prepare full-length cDNA (as already published) and ONT for long read sequencing, rather than a development of a new method, but they show that their approach can be successfully compared to other existing experimental and computational approaches.

Overall the paper is clearly written and well organized, and the data allows for some interesting findings regarding biology, specially regarding alternative splicing AS (i.e. the most abundant AS events are complex splicing changes, which are difficult to capture with short read sequencing. I do not have serious technical issues with the manuscript, but I have the following issues that the authors may want to take into account:

1.	The authors write "the throughput of current long-read sequencing platforms is still not comparable to short-read platforms and the per-base accuracy is also lower". PacBio Sequel II HiFI reads now match HiSeq in base accuracy

*Response: We thank reviewer 2 for their constructive feedback on our work. We agree that PacBio has higher accuracy, and have modified the sentence as follows to convey our thoughts more clearly:*

*‘the throughput of current long-read sequencing platforms is still not comparable to short-read platforms (especially for Pacific Biosciences Sequel platform) and the per-base accuracy may also be lower (particularly for Oxford Nanopore Technologies).’*

*We chose to apply nanopore sequencing in our study primarily because of its sequencing throughput advantage over PacBio.*

2.	The authors should comment on what gives FLAMES such a striking advantage over well-established tools. According to Fig S2 it reaches ~100% accuracy on SIRVs using ONT direct RNA data. This is really striking, but sensitivity and precision values should be provided as a supplementary table or the like.

*Response: When we started this project, our goal was to use an existing solution like FLAIR or TALON for isoform analysis, however we were dissatisfied with the results from these programs, which led us to develop FLAMES. FLAMES assumes there are truncated reads in the data and based on expression level, if one isoform is similar to a reference isoform but lacks the first exon then it is likely to be a truncated read instead a novel isoform. Our method is therefore more aggressive at collapsing isoforms that are highly similar to each other in terms of their 3’ / 5’ ends, which may help reduce the number of spurious isoforms called due to truncated reads and other artefacts. We believe that this step contributes to the improved performance of FLAMES relative to other methods.*

*As requested, we have added a new Table (S2, see below) that shows the number of discoveries each method makes in the different categories of interest (false negative, true positive and false positive) for the SIRV data.*

3.	Genome-mapped and SIRVome-mapped reads should undergo the same processing steps (as explained page 19, lines 58 onward for the former). More specifically, do the SIRV reads also undergo the "two-pass" polishing steps mentioned on page 20?

*Response: Yes, all datasets (SIRV, human and mouse) were processed in the same manner for the isoform detection and quantification step, using FLAMES’ default settings. The workflow is outlined in the top part of the new Supplementary Figure S2.*

4.	Figure S3. If I understand the figure, there is a strong 3' bias, which suggests that the transcripts may be rather short. Also, the bias is quite different for scimixology1 and scimixology2. Any hypothesis to explain this?

*Response: Yes - our hypothesis on this is mentioned in the Methods and is repeated below:*

*‘We noticed that the scmixology2 data contained more reads that were not full-length, which might relate to the difference in sample preparation time or the v2 and v3 10x chemistry.’*

5.	Other than the comparison with the SIRVs there is little characterization of the transcripts sequenced: the distribution of transcript lengths, the proportion of full lengh transcripts, the proportion of known and novel transcripts—although some numbers are given in the text, we do not know how many novel transcripts have been discovered through this work--, what fraction novel transcripts map to intergenic regions, and of those whether there is any indication of coding potential (for instance, using PhyloSCF). For annotated transcripts do we get novel exons (any potentially novel protein coding exon?) Also, it is not clear what transcript annotation was employed.

*Response: Gencode annotation was used (human hg38.v33 or mouse mm10.vM24, as mentioned in the Methods section). Figure 2A-C is intended to charactersize the transcripts found in out data. Figure 2B shows the number of isoforms in different categories (2A) both in terms of number and proportion. The number of novel isoforms varies depending on the threshold used in FLAMES (we ignore isoforms with fewer than 5 reads). Because most of the novel isoforms have very low expression, we hypothesized in the Discussion section that they are more likely to be splicing noise rather than isoforms that will generate novel functional protein. The coding potential for each isoform is available as part of the SQANTI2 results which can be accessed in the data (https://github.com/LuyiTian/FLTseq_data/tree/master/data in FLTSA_output > transcript_assembly_classification.txt.gz > coding column). However, we found that some of the coding potential predictions were inconsistent with our prior knowledge (i.e. a number of known coding isoforms were classified as non-coding) so did not highlight these results in the main text.*

6.	I also think that it would be interesting to see how many novel transcripts do we get per cell, and whether there is some saturation as we add novel cells.

*Response: As per our response above, we think that the novel category may contain a lot of noise, particularly for transcripts with low abundance, and so did not focus on these results too much. We have added a subsampling analysis (between 20% and 80% of the original number of long reads from the scmixology2 data) to look at the impact sequencing level has on the differential transcript usage results. As one would expect, we do find fewer isoforms when we start with fewer reads, but nonetheless, the majority of genes detected to have differential transcript usage (58%) are found in common between all subsampling analyses (refer to the new Figure S7 for these results).*

7.	More details on the FLAIR runs are needed. For example, what module was used, "align", "correct", "collapse", etc., or all of them? I am not 100% familiar with FLARI or TALON, but it seems to me that skipping the collapse step would give them an unfair disadvantage when compared to other tools that collapse by default (such as StringTie2 and, I guess, FLAMES), both in terms of transcript model and TSS accuracy (truncated reads have a much higher probability of being collapsed into a full-length one).

*Response: We ran FLAIR with all the recommended modules, which include the ‘align’, ‘correct’ and ‘collapse’ steps (we have elaborated on this in the Methods). All methods compared accept known GTF annotation files as input to help correct the splicing sites so they were all run in a fair, semi-supervised way for isoform detection.*

8.	The "two-pass" polishing step looks very convoluted to me (to be honest I'm not sure I fully understand it), and it's unclear what advantage it brings. The authors should provide a comparison of accuracy of transcript models with and without it, for example on SIRVs

*Response: We apologize for the confusion and have added a new Supplementary Figure (S2) to clarify and elaborate on the steps involved in different parts of the FLAMES analysis pipeline. We don't polish twice in the workflow, rather we align the reads in a two-pass manner. In the first pass, we align the long-reads to the genome and at this stage we polish and define the isoforms based on these results. The second alignment step aligns reads to the isoforms determined for the purpose of distinguishing between similar isoforms and quantification.*

9.	page 9, line 44: "Around 80% of genes can be expressed as multiple isoforms. " Please provide a reference for this statement

*Response: This statement refers to the results in Figure 2E. We have added mention of this panel to the end of the sentence above in our revised manuscript to make this clearer.*

10.	Figure 2F. "The distribution of isoforms expression is skewed with only a few abundant transcripts dominantly expressed for most genes." The authors should cite https://pubmed.ncbi.nlm.nih.gov/23815980/

*Response: Thank you for the reference, which we have added to the above sentence to note that the results shown in 2F are consistent with its findings.*

11.	The data on the different allele frequency of a few genes correlating with CLL expression clusters is quite interesting, but it is sort of frustrating that it is not bringing new biological insights. Maybe the authors could compare with the available DNA and matched bulk RNAseq data to see whether they could validate the sc results.

*Response: We thank the reviewer for the suggestion and have added a comparison that shows the significant variants detected in our single-cell analysis can also be identified in matched bulk RNA-seq (on sorted tumour cells) and DNA-seq (whole exome-sequencing). The updated results on this are as follows:*

*‘Material from the same sample was further analysed using bulk RNA-seq and whole exome sequencing. All four variants were detected in the bulk RNA-seq, and 3 out of 4 were present in the bulk whole exome sequencing (the RAD21 variant was outside the capture region). The variant in PHEX is a known polymorphism and the variant allele frequency was consistent with it being a germline SNV, so the difference of its allele frequency could be a result of allele-specific expression.’*

**Second round of review**

**Reviewer 1**

1. While the reviewer appreciates the inclusion of data that extends findings from the current individual example SF3B1 patient (reviewer only figures), it would be important to have some orthogonal validation for the single-patient as originally requested (statistical measures of sensitivity), which should be extremely easy for the authors using published bulk deep RNA-Seq short-read PSI data for associated isoform junctions (patient-specific junctions). While it is clear the approach is working (detecting some known events), how well is not clear (how sensitive is the approach for detecting bulk events).

2. Please clarify in the text that CD45 is the only example in which multiple antibodies targeting different isoforms existing in this analysis, as this will not be immediately obvious to most readers.

3. Supplemental Figure 6 shows evidence that long read sequencing facilitates greater sensitivity to detect new exon-exon junctions, versus bulk RNA-Seq. However, the author conclusion that long-reads inherently provide better detection of novel junctions is flawed. Rather, the increased sensitivity to detect novel isoforms in the long-read data is likely due to the increased purity of rare cell-type specific splicing variation. As such, this result should be explainable by considering which cell populations have the most novel isoforms.

**Reviewer 2**

The authors have succesully addressed my concerns. In my opinion, they have also done a good job in adressing the issues raised by reviewer 1!

**Authors Response**

**Point-by-point responses to the reviewers’ comments:**

Re-review comments:

Reviewer #1: In addition to clarifying the novelty and comprehension of the FLAMES workflow for genomic variant detection, the authors provide rationale and justification for most of the prior reviewer concerns. One remaining concern is the lack of comparison of differential isoforms to alternative splicing/promoter events from prior reasonably matching bulk RNA-Seq. These would provide quantitative insights into how well FLAMES does at detecting prior observed bulk events.

1. While the reviewer appreciates the inclusion of data that extends findings from the current individual example SF3B1 patient (reviewer only figures), it would be important to have some orthogonal validation for the single-patient as originally requested (statistical measures of sensitivity), which should be extremely easy for the authors using published bulk deep RNA-Seq short-read PSI data for associated isoform junctions (patient-specific junctions). While it is clear the approach is working (detecting some known events), how well is not clear (how sensitive is the approach for detecting bulk events).

*Response: We apologise for any confusion, but the current patient featured in our study (CLL2) does not have an SF3B1 mutation (the examples shown in the reviewer only figures were from a separate set of patients) so the type of validation suggested by the reviewer is not as straightforward as suggested unfortunately. We certainly take the point however that comparing the single-cell isoforms detected with PSI results from relevant deeply sequenced bulk short-read RNA-seq is a good form of orthogonal validation, and will aim to include such an analysis in our follow up study that includes these SF3B1 patients (we may need to generate matched bulk RNA-seq) or publicly available data.*

2. Please clarify in the text that CD45 is the only example in which multiple antibodies targeting different isoforms existing in this analysis, as this will not be immediately obvious to most readers.

*Response: We sincerely apologise for getting our wires crossed in our response to this question. As the ADT expression plot you requested clearly shows, CD45 does not have multiple antibodies targeting different isoforms. Rather it was the only gene from the panel of 17 CITE-seq antibodies for which differential transcript usage was detected in the single-cell data. This is the reason it was highlighted in the UMAP plot to show the correlation between a specific isoform and antibody expression in different cells. Sorry again for the confusion on this. We have added a note on this important detail to the Results section so that it is clear (i.e. ‘CD45 was the only DTU gene identified amongst the CITE-seq panel of 17 antibodies.’) We plan to use a much larger panel of antibodies (100+) to uncover more examples like this in future experiments.*

3. Supplemental Figure 6 shows evidence that long read sequencing facilitates greater sensitivity to detect new exon-exon junctions, versus bulk RNA-Seq. However, the author conclusion that long-reads inherently provide better detection of novel junctions is flawed. Rather, the increased sensitivity to detect novel isoforms in the long-read data is likely due to the increased purity of rare cell-type specific splicing variation. As such, this result should be explainable by considering which cell populations have the most novel isoforms.

*Response: Thank you for the comment. The UMAP in Figure S1A shows that each cell line is fairly homogeneous in terms of the cell types present, so we don’t think rare cell-type specific splicing variation is likely to be the reason for the difference. Also the junctions in the bulk data are examined separately for each cell-line (or patient for CLL2) i.e. junctions with greater than 5 uniquely mapping reads for the specific sample being compared are included in the analysis. In the single-cell data, at least 1 supporting read was required (in the relevant cell cluster for the cell line data) to account for the relatively smaller total library sizes from the single cell data compared to the bulk. Although we think the results have been matched up as carefully as possible to allow a fair comparison, we have removed the sentence that suggests that the long-read data is delivering improved sensitivity (i.e. deleted ‘This further demonstrates the benefit of long-read data for the detection of novel splicing events’) in case there is another explanation for the low degree of overlap of novel junctions unrelated to sensitivity.*
